# Personals

**Published:** 1913-09

**Authors:** 


### PERSONALS.

---

***Announcements of removals, travel, and other matters of interest are requested. Please report any errors in the listing of any physician, in the State and other directories that we may co-operate with the State Society in securing a correct list of physicians.***

---

Auburn City Medical Society elected Dr. Geo. W. Greene, President, Dr. O. B. Swayze, Vice-President, Dr. L. Belle Richens, Secretary-Treasurer, for year 1913-14.

---

Dr. T. M. Laurie of Auburn left August 4 for a three weeks' auto trip to include Long Island, the Berkshire and Green Mountain regions.

---

Dr. M. L. Seccomb of Auburn is spending a month in the Adirondacks.

---

Dr. Ellen R. Spragge, Buffalo, 1888, is located at 926 Academy avenue, St. Louis.

---

Dr. Charles G. Levesconte, Buffalo, 1888, of Kingscove, Bonavista Bay, New Foundland, writes us of a steamboat trip to attend a confinement case.

---

Dr. James Tyson, emeritus professor of medicine, University of Pennsylvania, recently passed the 50th anniversary of his graduation in medicine. The Northern Medical Association of Philadelphia presented him with a loving cup.

---

Dr. John M. Garratt announces the opening of his offices at 52 North Pearl St., Buffalo. Hours 8-11 and 1-3. X-ray diagnosis and treatment, High frequency and allied currents, comprise his special interests.

---

Dr. Maud J. Frye of Buffalo, spent part of August in the Adirondacks.

Dr. T. J. Hogan has moved from Attica to 29 Main street, Lockport.

---

Dr. G. W. E. Goodell has moved from Bridgeport to Wayland.

---

Dr. A. A. Jones of Buffalo sailed for Europe late in July.

---

Dr. Douglas P. Arnold of Buffalo has gone to Europe for a year's post-graduate study.

---

Dr. Mary B. Wetmore of Buffalo has moved her office and residence to 273 Woodlawn avenue.

---

Dr. Charles Meine of Germania, Pa., is postmaster of that town.

---

Dr. Jacob Goldberg of Buffalo, Chairman of the Board of School Examiners, has an interesting and conservative article on the teaching of sex hygiene to youths in *Truth* of July 26.

---

Dr. James S. Allen of Geneva is in Europe.

---

Dr. Frederick A. Hayes of Buffalo spent the first half of August motoring through the Catskills and along the Jersey coast.

---

Dr. A. G. Bennett of Buffalo and Dr. Wm. D. Johnson of Batavia were the speakers at the summer meeting of the Genesee Co. Medical Society.

---

Dr. A. E. Woehnert of Buffalo has returned from Europe.

---

Dr. Dewitt H. Sherman of Buffalo spent part of August in Newport and New Hampshire.

---

Dr. Warren W. Britt has removed his office to the residence of the late Dr. F. F. Hoyer, 32 Grove street, Tonawanda, N. Y.

